# Characteristics and significance of programmed cell death‐related gene expression signature in skin cutaneous melanoma

**DOI:** 10.1111/srt.13739

**Published:** 2024-05-20

**Authors:** Xiaoxia Wu, Suhong Chen, Qingfa Ji, Han Chen, Xiuxia Chen

**Affiliations:** ^1^ Department of Dermatology The 95th Hospital of Putian Putian Fujian China; ^2^ Department of Dermatology Putian First Hospital of Fujian Province Putian Fujian China; ^3^ Department of Dermatology Putian City Dermatology Prevention and Treatment Hospital Putian Fujian China; ^4^ Laboratory Pathology Department Joint Logistics Support Force 900th Hospital Cangshan Campus Fuzhou Fujian China; ^5^ Department of Anesthesiology The 95th Hospital of Putian Putian Fujian China

**Keywords:** drug sensitivity, melanoma, programmed cell death, survival, tumor microenvironment

## Abstract

**Background:**

Programmed cell death (PCD) pathways play crucial roles in the pathogenesis of skin cutaneous melanoma (SKCM). Understanding their prognostic significance and clinical implications is imperative for the development of personalized treatment strategies.

**Methods:**

A total of 1466 PCD‐related genes were analyzed using data from The Cancer Genome Atlas (TCGA)‐SKCM cohort (*n* = 353). Prognostic cell death index (CDI) was established and validated through survival analysis and predictive modeling. Functional enrichment, protein‐protein interaction (PPI), consensus clustering, and tumor microenvironment assessment and drug sensitivity analysis were performed to elucidate the biological and clinical relevance of CDI.

**Results:**

CDI effectively stratified SKCM patients into high and low‐risk groups, demonstrating significant differences in survival outcomes. It exhibited predictive value for survival at 1, 3, and 5 years. The concordance index (C‐index) was 0.794 in the training set, and 0.792 and 0.821 in the internal and external validation sets, respectively. The corresponding area under curve (AUC) was all above 0.75 in these data sets. Functional enrichment analysis revealed significant associations with immune response and inflammatory processes. PPI analysis identified key molecular modules associated with apoptosis and chemokine signaling. Consensus clustering unveiled three discernible subtypes demonstrating notable disparities in survival outcomes based on CDI expression profiles. Assessment of the tumor microenvironment highlighted correlations with immune cell infiltration such as M1 macrophages and T cells. Drug sensitivity analysis indicated tight correlations between CDI levels and response to immunotherapy.

**Conclusion:**

Our comprehensive analysis establishes the prognostic significance of PCD‐related genes in SKCM. CDI emerges as a promising prognostic biomarker, offering insights into tumor biology and potential implications for personalized treatment strategies. Further validation and clinical integration of CDI are warranted to improve SKCM management and patient outcomes.

## 1 INTRODUCTION

Programmed cell death (PCD) is a crucial fundamental process for maintaining tissue homeostasis and eliminating damaged or unnecessary cells in multicellular organisms.^1^ In contrast to necrosis, an uncontrolled and passive mechanism of cell death often triggered by external factors such as physical trauma or toxins, PCD represents a meticulously regulated and structured process orchestrated by specific molecular pathways intrinsic to cellular function.^2^ PCD encompasses various forms and mechanisms, each serving distinct biological functions. Apoptosis, the most well‐characterized form of PCD, involves a series of tightly regulated molecular events leading to cell shrinkage, chromatin condensation, DNA fragmentation, and ultimately the formation of apoptotic bodies that are efficiently engulfed by neighboring cells or phagocytes.^3^, ^4^ Another type of PCD, known as autophagy, plays a critical role in preserving cellular homeostasis by facilitating the degradation and recycling of cellular components, particularly under conditions of nutrient scarcity or stress.^5^, ^6^ In addition to apoptosis and autophagy, other forms of PCD, such as pyroptosis, necroptosis, and ferroptosis, have emerged as important players in various physiological and pathological contexts.^7^ Pyroptosis, for instance, is a pro‐inflammatory form of cell death triggered by activation of the inflammasome, leading to rapid cell swelling and lysis accompanied by the release of inflammatory mediators.^8^ Necroptosis, on the other hand, exhibits characteristics akin to necrosis but is genetically programmed and regulated by specific signaling pathways, notably implicating receptor‐interacting protein kinases (RIPKs) and mixed lineage kinase domain‐like protein (MLKL).^9^, ^10^ Overall, PCD represents a diverse array of cellular processes with critical implications for development, tissue homeostasis, immunity, and disease pathogenesis. Enhanced comprehension of the intricate regulatory mechanisms underlying PCD is essential for developing therapeutic strategies targeting aberrant cell death pathways across diverse pathological conditions.

PCD also plays a pivotal role in the pathogenesis of skin cutaneous melanoma (SKCM), a malignant neoplasm originating from melanocytes in the skin. In SKCM, the dysregulation of PCD pathways significantly contributes to multiple facets of tumorigenesis, encompassing tumor initiation, progression, and therapeutic responsiveness.^11^, ^12^ Apoptosis is particularly relevant in SKCM, since alterations in the expression of pro‐apoptotic and anti‐apoptotic proteins disrupt the delicate balance between cell survival and death, promoting tumor cell evasion of programmed death and facilitating tumor growth and metastasis.^13^, ^14^ Moreover, mounting evidence suggests the implication of alternative PCD mechanisms in SKCM, including pyroptosis and ferroptosis.^15^, ^16^ Pyroptosis has been implicated in promoting inflammation and fostering the progression of SKCM.^17^, ^18^ Similarly, ferroptosis, characterized by iron‐dependent lipid peroxidation and accumulation of reactive oxygen species (ROS), has garnered attention for its role in SKCM pathogenesis.^19^ The dysregulation of ferroptosis‐related pathways, including disturbances in iron metabolism and lipid peroxidation, may potentially underlie the progression of SKCM and the emergence of therapeutic resistance.^20^, ^21^ Targeting dysregulated PCD pathways holds promise for the treatment of SKCM and overcoming therapeutic resistance, representing a significant area of investigation in cancer research.

Delving into the expression characteristics of PCD‐related genes in SKCM is crucial for understanding the intricate interplay between tumor biology and clinical outcomes. PCD dysregulation has been implicated in various aspects of SKCM pathogenesis, including the tumor's immune microenvironment, drug sensitivity, and prognosis. The altered PCD pathways in SKCM play a pivotal role in modulating immune microenvironment and influencing tumor progression.^20^ Cell death could release tumor‐derived neoantigens that are potentially capable of activating immune cells and triggering anti‐tumor immune reactions.^22^, ^23^ On the other hand, immune cell infiltration, cytokine signaling, and tumor‐immune cell interactions contribute to the regulation of PCD in SKCM. For instance, immune cells such as tumor‐infiltrating lymphocytes (TILs) and dendritic cells can either promote or suppress PCD in SKCM, depending on their functional phenotypes and activation status.^24^ Additionally, cytokines and chemokines produced by immune cells possess the capability to modulate PCD signaling pathways within the tumor microenvironment.^10^, ^25^ Moreover, the relationship between PCD dysregulation and drug sensitivity in SKCM is of significant clinical relevance. Alterations in PCD pathways can confer resistance or sensitivity to various therapeutic agents commonly used in SKCM treatment, including chemotherapy, targeted therapy, and particularly immunotherapy. Therefore, elucidating the molecular mechanisms underlying PCD‐mediated drug resistance or sensitivity is essential for developing more effective treatment strategies and overcoming therapeutic resistance in SKCM patients. Despite the increasing recognition of the significance of PCD in SKCM, there remains a dearth of comprehensive studies investigating its expression characteristics and clinical ramifications. The existing literature highlights the need for more detailed and integrated research efforts to decipher the complexities of PCD dysregulation in SKCM and its impact on patient outcomes. Elucidating the specific roles of PCD‐related gene expression signatures in SKCM pathogenesis will be critically important for the development of novel prognostic markers and therapeutic interventions tailored to individual patient's needs.

The aim of this study is to delineate the expression characteristics of PCD‐related genes in SKCM and elucidate its clinical implications. By meticulously explicating the genetic landscape associated with PCD dysregulation in SKCM, this study discerns the key molecular pathways implicated in melanoma pathogenesis. Understanding these intricate features holds promise for unraveling the underlying mechanisms governing melanoma initiation and progression. Additionally, this study seeks to elucidate the significance of PCD‐related gene expression signature in identifying novel biomarkers and therapeutic targets, thereby advancing our understanding of SKCM pathophysiology and expediting the development of more effective treatment modalities.

## 2 METHODS

### 2.1 Study design

A total of 1466 genes associated with twelve types of PCD were collected from previous literature,^26^ which collected the genes associated with different types of PCD from gene sets of GSEA, KEGG pathways, review papers, and manual collation. The dysregulated PCD is usually involved in various physiological and pathological processes, not limited to a single disease or cancer, thus the genes from the previous literature were also applicable in this study. Utilizing the expression matrix and survival information of patients from The Cancer Genome Atlas (TCGA)‐SKCM cohort, PCD‐related genes with prognostic value were screened to construct a prognostic cell death index (CDI). Subsequently, internal and external validation of CDI's prognostic performance was conducted. Upon confirmation of the prognostic significance of CDI, further investigations were performed to explore its association with clinical outcomes, functional enrichment, protein‐protein interaction (PPI), consensus clustering, tumor microenvironment, and drug sensitivity.

### 2.2 Data preprocessing

The raw data of RNA sequencing for the TCGA‐SKCM cohort was initially obtained via the Genomic Data Commons (GDC) platform (https://portal.gdc.cancer.gov/)^27^ in count format. In instances where different probes were utilized for the detection of the same gene within the RNA sequencing data, the average expression level of each corresponding gene was calculated. To ensure data quality for subsequent analysis, filtering procedure on the expression data was conducted. This entailed the exclusion of missing values and the removal of genes with low detection levels (count < 1). In order to mitigate batch effects arising from variations in sequencing among different patients, batch effect correction on the sequencing data was performed using the limma package in R software, followed by data normalization procedures. The same methodology was applied to the RNA sequencing data of the SKCM cohort from the GSE98394 dataset in the Gene Expression Omnibus (GEO), which served as the validation set.

### 2.3 Establishment and validation of prognostic CDI

To identify PCD‐related genes correlated with the survival of SKCM patients, 70% of patients from the TCGA‐SKCM cohort were randomly selected as the training set. The univariate Cox regression analysis used each subject's overall survival, defined by survival duration and status, as the dependent variable. Expression levels of PCD‐related genes were treated as continuous independent variables. Cox models were built, calculating regression coefficients, hazard ratios, 95% confidence intervals, and statistical significance (*p*‐values) for each gene. Due to the significant number of prognostic genes (*p* < 0.05) from the univariate analysis, LASSO analysis was applied to reduce variables. The “glmnet” package in R software performed LASSO analysis with 10‐fold cross‐validation, using “cox” as the “family” parameter. The most suitable lambda value, determined by cross‐validation, was used to finalize the penalized variables. Further variable selection was carried out using the “step” function (backward) of the “rms” package in R, following the establishment of a multivariate Cox regression model based on these variables. The optimal multivariate Cox regression model, selected using the stepwise variable selection method, was chosen based on the Akaike Information Criterion for best fit. Based on the expression levels of PCD‐related genes in the prognostic signature, CDI was calculated using the following formula: CDI = coefficient 1 × (level of PCD gene 1) + coefficient 2 × (level of PCD gene 2) + …… + coefficient n × (level of PCD gene n).

To assess the predictive efficacy of CDI in terms of survival, the remaining 30% of patients were used as the internal validation set, while the GSE98394 dataset containing survival data and expression data of SKCM patients was utilized as the external validation set. In the training set, internal validation set, and external validation set, CDI for each SKCM patient was calculated. Patients were sorted into high‐CDI and low‐CDI categories based on specific demographics. In the initial assessment of the training set, individuals were divided into high‐ and low‐CDI groups using the median CDI score of the entire training cohort. Those exceeding the median CDI score were placed in the high CDI category, while those below were placed in the low CDI category. Additionally, for the validation stage, the high and low CDI categories were determined by the median CDI score of the validation set. A comparison of the overall survival Kaplan‐Meier curves for the high and low CDI groups was conducted using the log‐rank test, with statistical significance set at *p* < 0.05. Receiver operating characteristic (ROC) curves were generated using CDI score as predictive variable for 1‐year, 3‐year, and 5‐year survival, and the area under the curve (AUC) was calculated. Calibration curves and dot plots were utilized to assess the concordance between CDI‐predicted survival and actual survival.

### 2.4 Association between CDI and clinical features

All patients from TCGA‐SKCM were included in this analysis. The association between patients' CDI and the subsequent clinical features was analyzed, including: survival status, age, gender, race, TNM stage, the American Joint Committee on Cancer (AJCC) stage, and prior treatments.

### 2.5 Functional enrichment analysis

In order to elucidate the variances in biological functions between SKCM patients with high CDI and low CDI, the expression level differences of all genes between high CDI and low CDI patients were compared. Genes exhibiting a statistical significance with a *p*‐value < 0.05 and |log2FC| > 2 were selected as differentially expressed genes (DEGs).

Functional enrichment analysis based on DEGs was conducted to explore the biological functions associated with CDI. Firstly, the DEG gene list was imported into the Database for Annotation, Visualization, and Integrated Discovery (DAVID),^28^ and online analysis was performed to identify biological signaling pathways enriched in Gene Ontology (GO) and Kyoto Encyclopedia of Genes and Genomes (KEGG). To further validate these changes in biological functions, Metascape (https://metascape.org/)^29^ online tool was utilized for enrichment analysis of DEGs.

### 2.6 PPI analysis

To analyze the interactions among proteins coded by the modeling genes of PCD signature and DEGs, these genes were inputted into online tools such as Metascape,^29^ STRING (Search Tool for the Retrieval of Interacting Genes/Proteins),^30^ and GeneMANIA^31^ for comprehensive PPI analysis.

### 2.7 Unsupervised consensus clustering

Unsupervised consensus clustering analysis was undertaken utilizing the DEG expression matrix of TCGA‐SKCM patients to elucidate unrecognized subtypes within this cohort. Parameters for consensus clustering were configured as follows: maxK = 8, clusterAlg = hc, distance = pearson. Visualization was conducted to delineate the distribution of survival status and CDI within the resultant clusters, culminating in the generation of Kaplan‐Meier curves for the three identified subtypes to evaluate disparities in survival outcomes.

### 2.8 Tumor microenvironment assessment

A gene set comprising 70 immune regulatory modulators was collected from previous literature,^32^ encompassing functions such as antigen presentation, cell adhesion, co‐inhibitors, co‐stimulators, ligands, receptors. The correlation between the expression levels of these genes in TCGA‐SKCM patients and CDI was investigated. Subsequently, CIBERSORT^33^ was utilized to evaluate the infiltration of 22 immune cell types in tumor samples of TCGA‐SKCM patients, including DC cells, M0, M1, M2 macrophages, T cells, B cells, and so forth and their association with CDI was analyzed. The most correlated immune cells were chosen, whereby patients were subsequently stratified into high and low abundance groups based on the median level of immune cell infiltration. Kaplan‐Meier survival curves and Log‐Rank tests were executed to ascertain the impact of these immune cells on survival outcomes.

### 2.9 Drug sensitivity prediction

Based on the expression profiles of TCGA‐SKCM patients, oncoPredict package^34^ was utilized to predict and calculate the IC50 values for 198 common anti‐cancer drugs for each patient. To forecast potential response to immunotherapy, the Tumor Immune Dysfunction and Exclusion (TIDE) method^35^, ^36^ was employed to estimate the response to immunotherapy, TIDE score, T‐cell dysfunction score, and T‐cell exclusion score for each TCGA‐SKCM patient.

### 2.10 Statistical analysis

All statistical analyses in this study were performed using R software (version 4.3.0). Survival analysis was conducted using the survival, survminer, and ggrisk packages in R. LASSO regression analysis utilized the glmnet package, while multivariable COX analysis and Stepwise model selection were carried out using the rms package. ROC curve plotting and AUC calculation were performed using the pROC and timeROC packages, while calibration curves utilized the car and rms packages. Calibration dot plots were generated using the PredictABEL package. Heatmap plotting was done using the pheatmap package. Differential gene expression analysis employed the limma package, and unsupervised clustering utilized the ConsensusClusterPlus package. Analysis of the tumor microenvironment involved the use of the CIBERSORT package, while drug sensitivity analysis utilized the oncoPredict package. A *p*‐value less than 0.05 was considered statistically significant.

## 3 RESULTS

### 3.1 The establishment of prognostic CDI in SKCM

**FIGURE 1:**
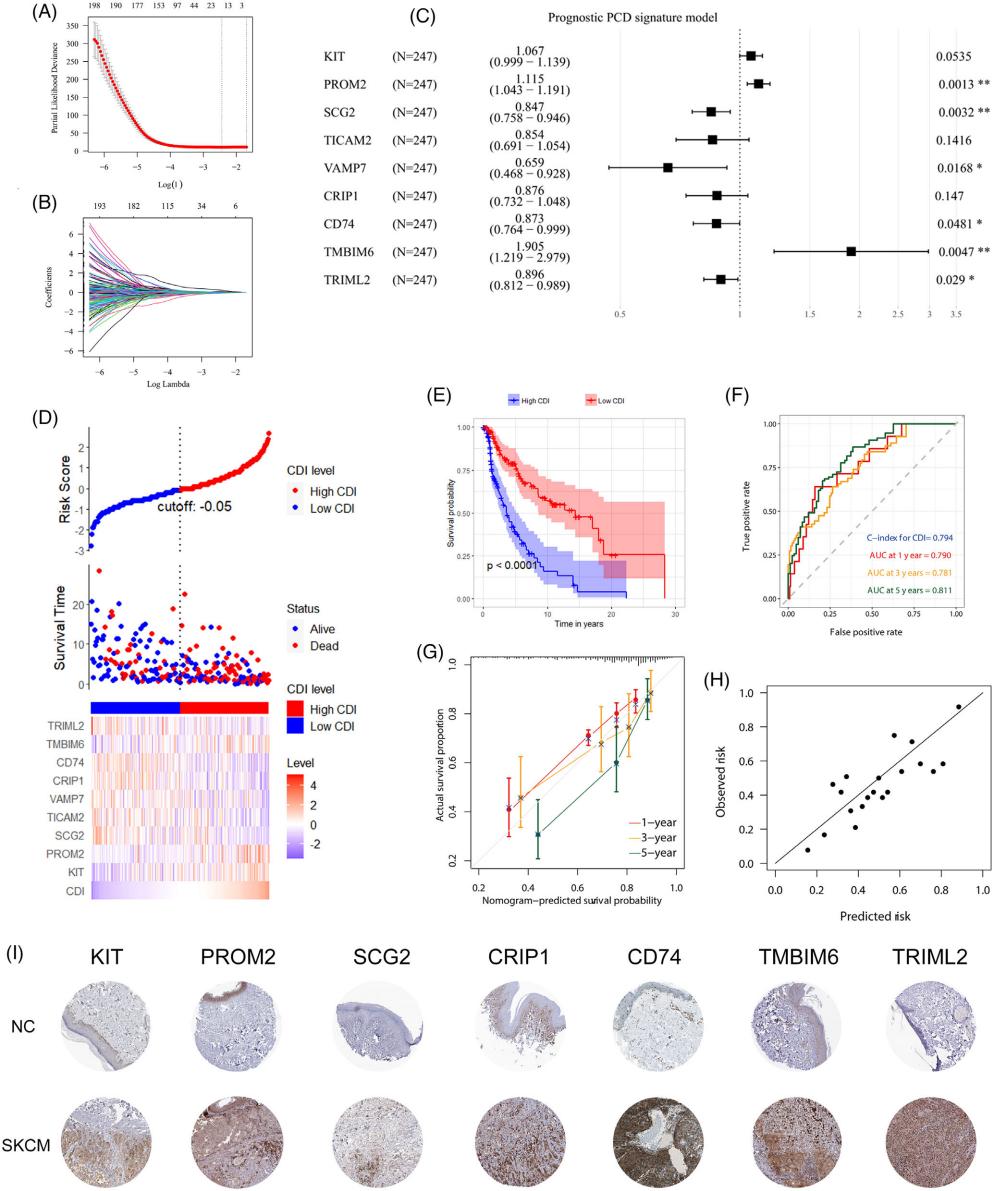
Establishment of prognostic CDI in SKCM. (A) Plot showing the variation of mean square error with Log(λ) in Lasso regression. (B) Curve depicting the change in regression coefficients with Log(λ) in Lasso regression. (C) Forest plot illustrating the HR values, 95% CI, and P‐values of each gene in the multivariable model. (D) Distribution of survival status for CDI. (E) Kaplan‐Meier curves comparing survival between high and low CDI groups. (F) ROC curves and corresponding AUC for CDI as a predictive variable at 1, 3, and 5 years. (G). Calibration curves for CDI as a predictive variable at 1, 3, and 5 years. (H). Dot plot representing the calibration of CDI as a predictive variable at 1, 3, and 5 years. (I) The IHC staining of the prognostic genes‐encoded proteins in the normal skin and in SKCM. AUC, area under curve; CDI, cell death index; IHC; immunohistochemical; ROC, receiver operating characteristic; SKCM, skin cutaneous melanoma.

In the training set, a total of 16 PCD‐related genes were identified through LASSO regression, penalizing for collinearity of the genes screened by univariate COX analysis (Figure 1A and 1B). The final model derived through Stepwise method in multivariate COX regression analysis comprised nine genes (Figure 1C), among which six were independent prognostic factors (*p* < 0.05), while the other three exhibited trends towards statistical significance in the multivariate COX model. Among the nine genes in the model, three were identified as risk genes, while the remaining six were protective genes.

In the training set, a higher proportion of death events was observed in SKCM patients with high CDI, with shorter survival time compared to those with low CDI, as determined by visual assessment (Figure 1D). The survival curves of patients in high and low CDI groups showed significant deviation (Figure 1E), indicating a notable survival difference (*p* < 0.001). Utilizing CDI as a predictive variable yielded a C‐index of 0.794, and an excellent predictive performance for survival at 1/3/5 years with corresponding AUCs of 0.79, 0.781, and 0.811 (Figure 1F). Calibration curves demonstrated good alignment between predicted and actual survival rates at 1/3/5 years (Figure 1G), further supported by calibration dot plot showing favorable concordance (Figure 1H). In these nine prognostic PCD genes, the immunohistochemical (IHC) staining of seven genes are documented in the Human Protein Atlas database. The IHC staining results of these seven genes are presented in Figure 1I, demonstrating that the majority of these genes show significantly elevated expression in SKCM, while they are scarcely expressed in normal skin. This further suggests the functional role of these genes at the protein level in SKCM.

### 3.2 Validating the predictive performance of CDI in internal and external validation sets

**FIGURE 2:**
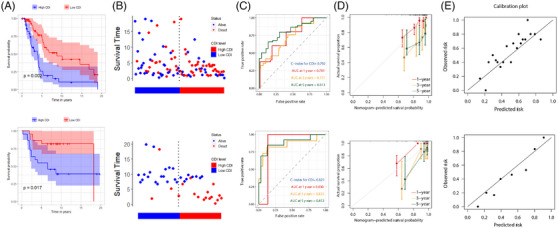
Validation of the predictive performance of CDI in internal and external validation sets. (A) Kaplan‐Meier curves comparing survival between high and low CDI groups. The upper panel represents the internal validation set, while the lower panel represents the external validation set. (B) Distribution of survival status for CDI. The upper panel represents the internal validation set, while the lower panel represents the external validation set. (C) ROC curves and corresponding AUC for CDI as a predictive variable at 1, 3, and 5 years. The upper panel represents the internal validation set, while the lower panel represents the external validation set. (D) Calibration curves for CDI as a predictive variable at 1, 3, and 5 years. The upper panel represents the internal validation set, while the lower panel represents the external validation set. (E) Dot plot representing the calibration of CDI as a predictive variable at 1, 3, and 5 years. The upper panel represents the internal validation set, while the lower panel represents the external validation set. AUC, area under curve; CDI, cell death index; ROC, receiver operating characteristic.

In both the internal and external validation sets, CDI effectively stratified patients into high and low‐risk groups, as demonstrated by distinctly separated Kaplan‐Meier curves (Figure 2A), thereby highlighting significant disparities in survival times (both *p* < 0.05). A higher frequency of death events was observed among patients with high CDI (Figure 2B). As a prognostic variable, CDI demonstrated C‐indices of 0.792 and 0.821 in the two datasets, respectively. ROC curves for 1/3/5 years displayed favorable predictive performance (Figure 2C). Calibration curves (Figure 2D) and dot plots (Figure 2E) further confirmed the good alignment between CDI‐predicted and actual survival rates.

### 3.3 Exploring the association between CDI and clinical features

**FIGURE 3:**
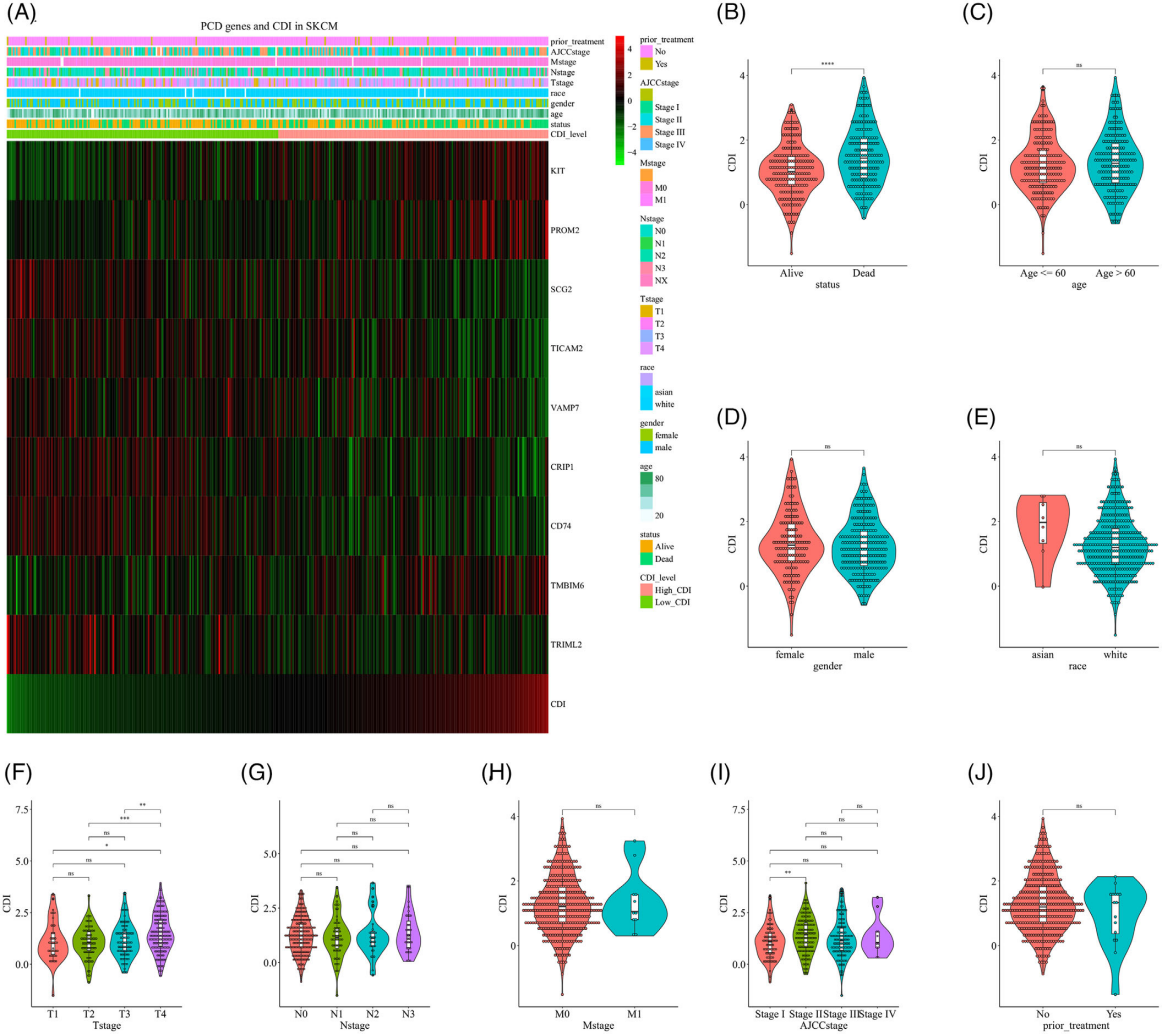
Exploration of the association between CDI and clinical features. (A) Relationship between CDI and the distribution of clinical features. (B) Association between CDI and survival status. (C) Association between CDI and age. (D) Association between CDI and gender. (E) Association between CDI and race. (F) Association between CDI and T stage. (G) Association between CDI and N stage. (H) Association between CDI and M stage. (I) Association between CDI and AJCC stage. (J) Association between CDI and prior treatment. CDI, cell death index.

The association between CDI and the distribution of clinical features is illustrated in Figure 3A. The mean CDI was significantly higher in deceased patients compared to surviving patients (Figure 3B), while there was no significant relationship observed between CDI and age (Figure 3C), gender (Figure 3D), or race (Figure 3E). With the advancement of T stage, CDI levels showed a proportional escalation (Figure 3F), whereas there was no significant association observed between CDI and N stage (Figure 3G) or M stage (Figure 3H). Regarding AJCC stage, CDI was significantly higher in stage II patients compared to stage I, while there was no significant variation observed in stages III and IV (Figure 3I). The preceding treatment history exhibited no discernible impact on CDI levels (Figure 3J).

### 3.4 Biological functional changes associated with CDI

**FIGURE 4:**
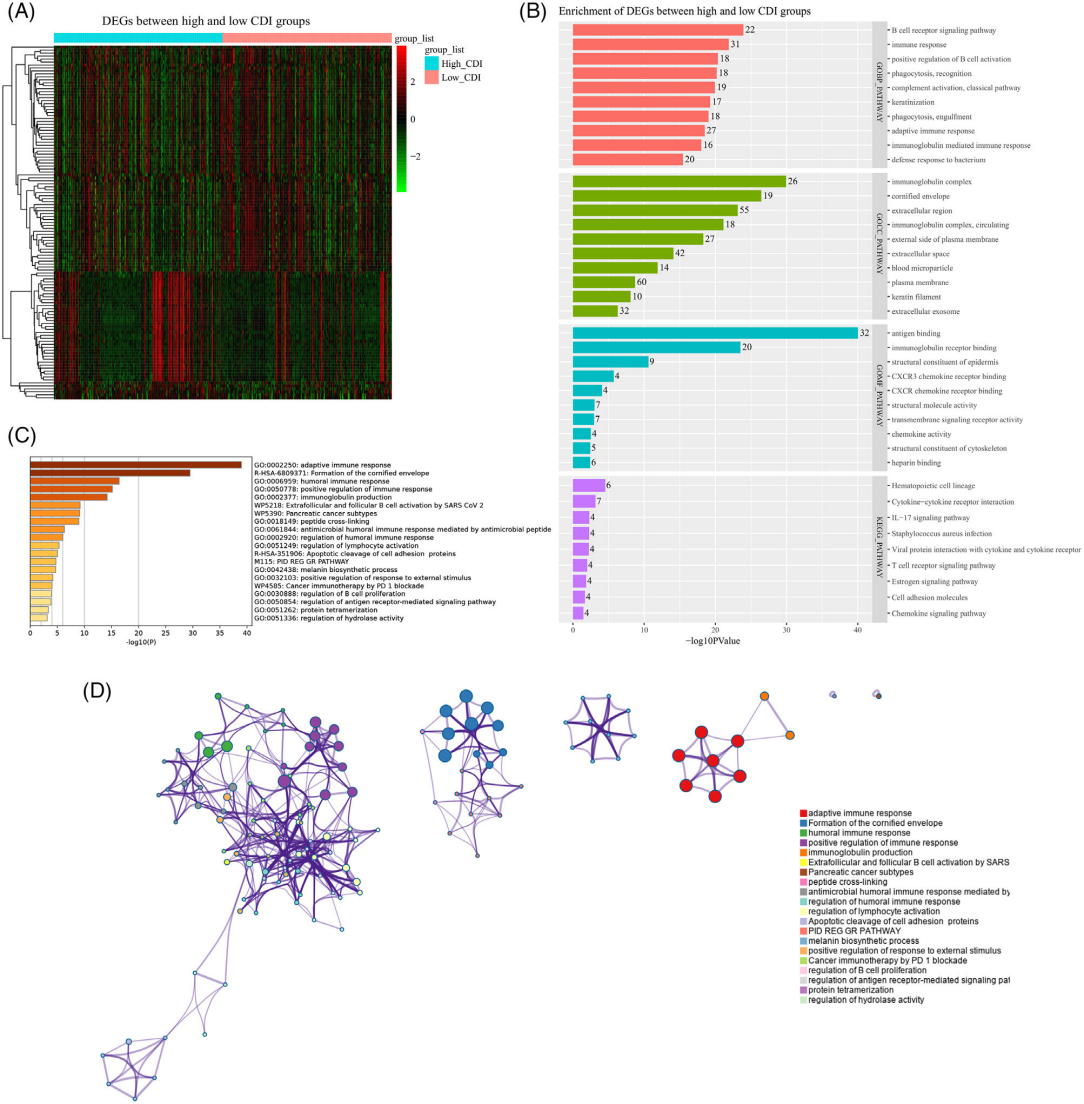
Biological functional changes associated with CDI. (A) Expression characteristics of differentially expressed genes (DEGs) observed between patients with high CDI and low CDI. (B) Enrichment analysis of DEGs conducted using the DAVID online tool. (C) Enrichment analysis of DEGs conducted using the Metascape online tool. (D) Network analysis of enriched pathways of DEGs conducted using the Metascape online tool. CDI, cell death index; DAVID; database for annotation, visualization, and integrated discovery; DEGs, differentially expressed genes.

In TCGA‐SKCM patients, significant differences in the expression characteristics of DEGs were observed between patients with high CDI and low CDI (Figure 4A). Functional enrichment analysis conducted employing the DAVID tool based on these identified DEGs unveiled substantial disparities in immune response and inflammatory processes between patients with high and low CDI. These includes B cell receptor, B cell activation, adaptive immunity, immunoglobulins, chemokines, and significant variations in phagocytosis function (Figure 4B). Furthermore, functional enrichment analysis conducted using Metascape also indicated significant alterations in immune responses, particularly concerning B cells, between the two patient groups (Figure 4C). Additionally, these DEGs were intricately linked with the WP4585 pathway of tumor anti‐PD1 immunotherapy. Moreover, these immune responses, B cells, apoptosis, and other signaling pathways exhibit complex interaction networks (Figure 4D).

### 3.5 PPI network associated with CDI

**FIGURE 5:**
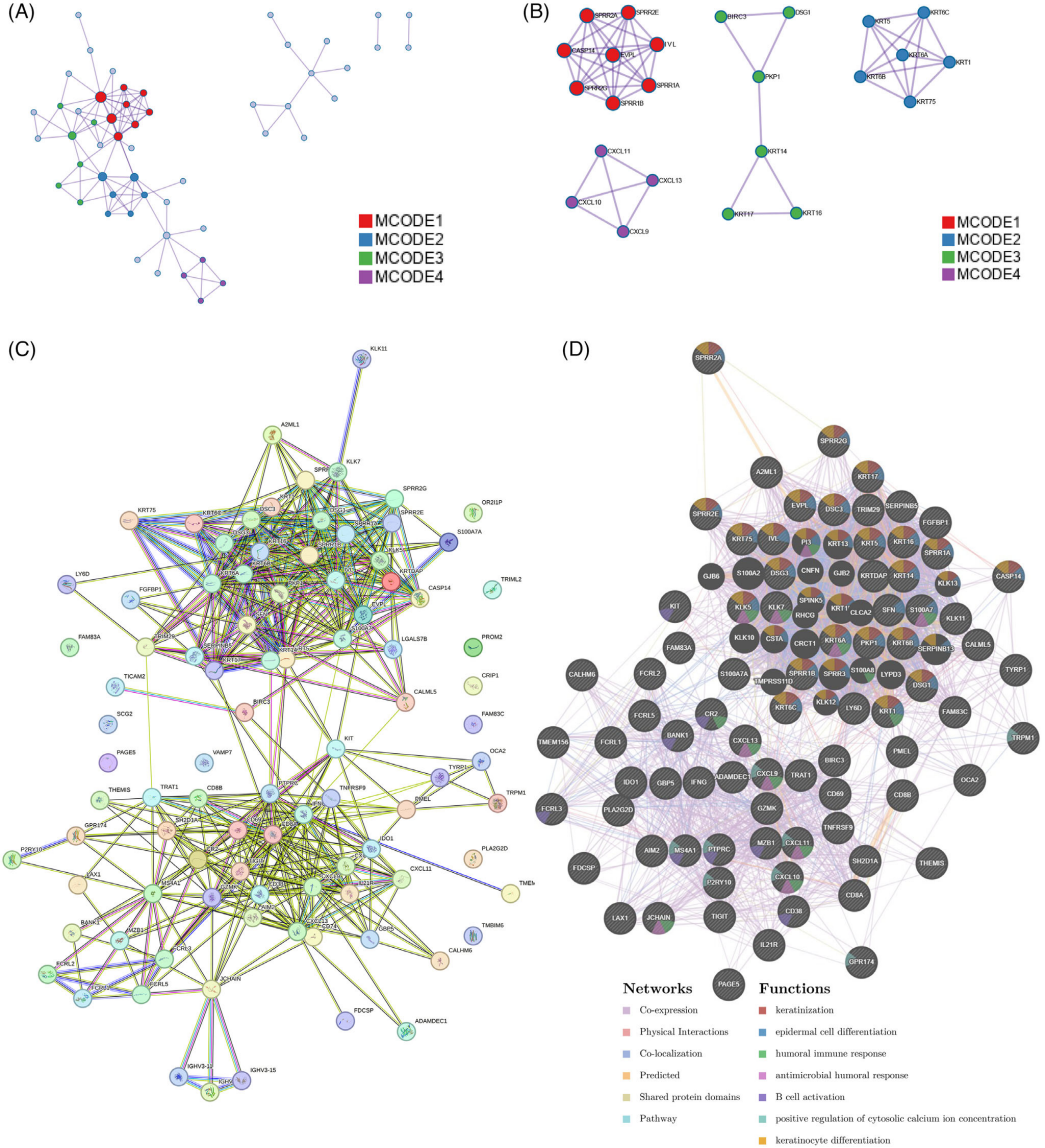
PPI Network associated with CDI. (A) and (B) Within the PPI network, four MCODE core modules were identified. (C) and (D) Complete PPI networks constructed using the STRING online tool and GeneMANIA online tool. CDI, cell death index; PPI, protein‐protein interaction; STRING, search tool for the retrieval of interacting gene.

Within the PPI network, four Molecular Complex Detection (MCODE) core modules were identified, as depicted in Figure 5A and 5B. These modules include the red module consisting of an interaction network of apoptosis‐related gene CASP14 and cornified cell envelope‐related small proline‐rich protein family genes, the purple module encompassing the network of four CXCL family chemokines, the green module representing the interaction network of apoptosis gene BIRC3 and genes associated with skin cells, and the blue module primarily composed of genes from the keratin gene family. The majority of genes situated within these core modules are located in central hub regions of the comprehensive PPI networks (Figure 5C and Figure 5D), indicating intimate interactions with proteins encoded by DEGs.

### 3.6 Unsupervised consensus clustering and CDI

**FIGURE 6:**
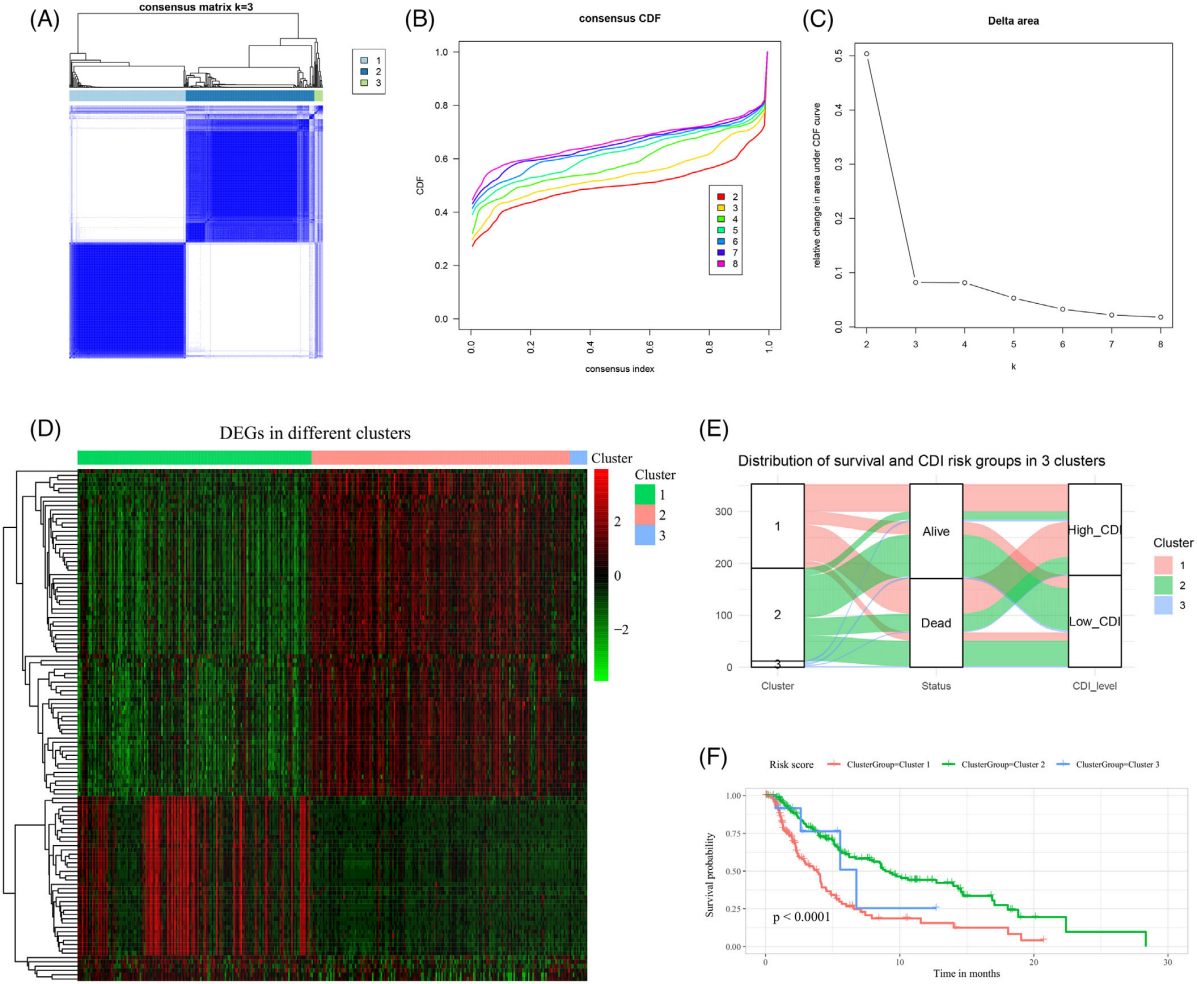
Unsupervised consensus clustering and CDI. (A) Unsupervised clustering matrix. (B) Cumulative distribution function (CDF) curve. (C) Area under the CDF curve. (D) Heatmap of expression matrix for all differentially expressed genes (DEGs) among patients in three clusters. (E) Distribution of survival status and CDI levels among patients in three clusters. (F) Kaplan‐Meier curves for survival among patients in three clusters. CDF, cumulative distribution function; CDI, cell death index; DEGs, differentially expressed genes.

The expression profiles of DEGs, as depicted in the heatmap (Figure 4A), unveiled a spectrum of the expression characteristics of DEGs among patients within both the high CDI and low CDI groups. This suggests the presence of clustering patterns beyond CDI based on the expression profiles of DEGs. Through unsupervised consensus clustering analysis, distinct expression patterns of DEGs were observed when clustered into three clusters (Figure 6A). Additionally, changes in the CDF curve (Figure 6B) and the area under the curve (Figure 6C) indicated that clustering into three clusters yielded the most significant and optimized clustering outcome. The heatmap depicting the expression profiles of DEGs across three clusters displayed remarkable consistency in expression characteristics within each cluster (Figure 6D). Notably, Cluster 1 and Cluster 2 encompassed a larger number of patients, while Cluster 3 consisted a smaller subset of patients. Cluster 1 exhibited a higher incidence of death events and elevated CDI (Figure 6E), and patients in Cluster 1 had significantly worse survival compared to those in Cluster 2 and 3 (Figure 6F).

### 3.7 Association of CDI with the immune microenvironment of SKCM

**FIGURE 7:**
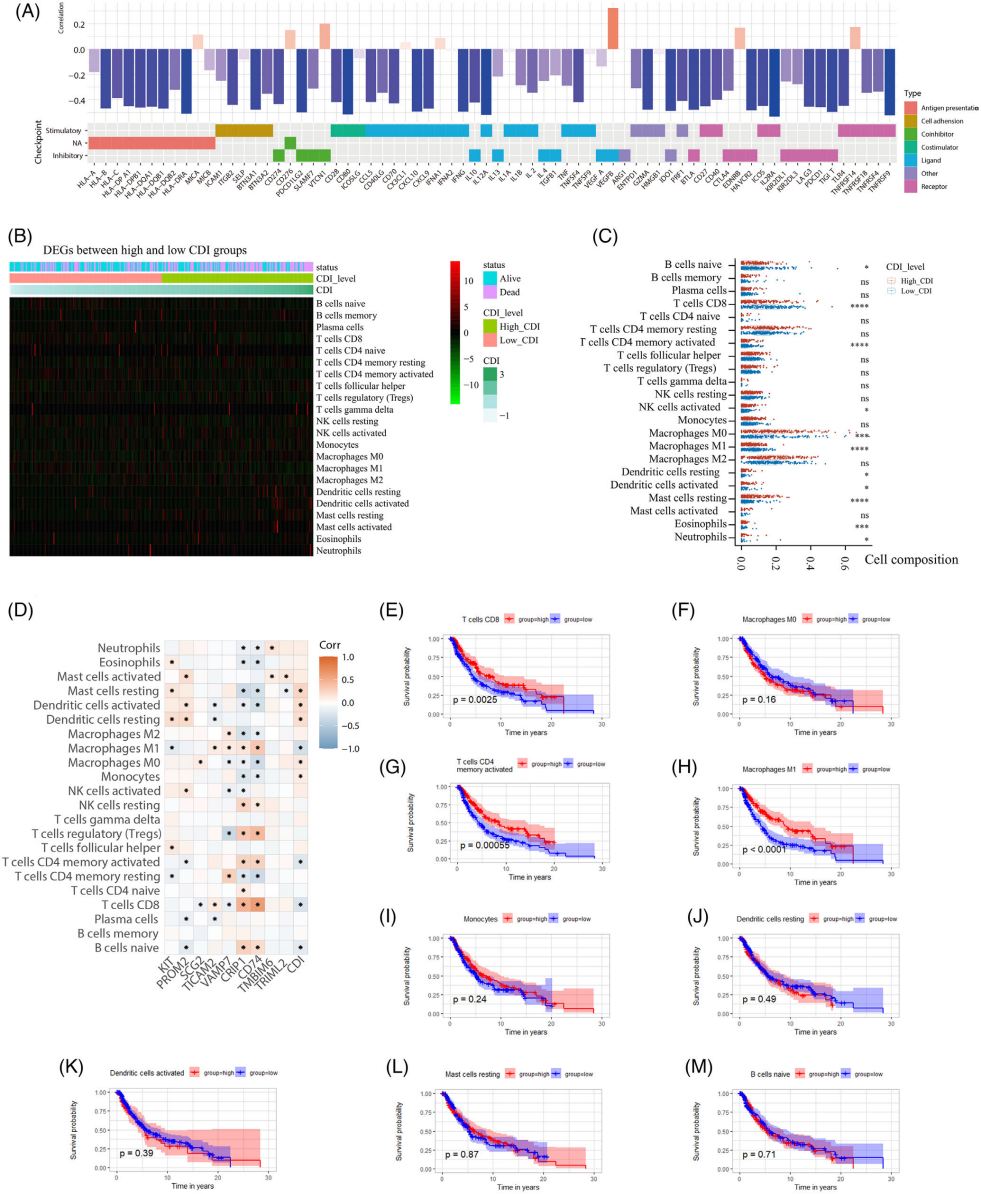
Association of CDI with the immune microenvironment of SKCM. (A) Correlation between the expression levels of 70 immune regulatory modulators and CDI. (B) Heatmap depicting the distribution of CDI with the infiltration levels of 22 immune cells. (C) Bar plot illustrating the differences in levels of 22 immune cells between patients with high CDI and low CDI. (D) Heatmap showing the correlation between CDI, modeling genes, and the levels of 22 immune cells, with asterisks indicating statistically significant correlations. (E–M) Kaplan‐Meier curves depicting the survival of patients with high and low levels of immune cells significantly associated with CDI. CDI, cell death index; SKCM, skin cutaneous melanoma.

Analysis of the correlation between the expression levels of 70 immune regulatory modulators and CDI revealed that approximately 90% of these modulators exhibited a negative correlation with CDI (Figure 7A). This suggests that patients with higher CDI levels may be in a state of immune suppression or immunodeficiency. The distribution of CDI with the infiltration levels of 22 immune cells is illustrated in Figure 7B. Quantitative analysis indicated that in comparison to patients with low CDI, those with high CDI demonstrated significantly elevated levels of activated NK cells, M0 macrophages, resting dendritic cells, activated dendritic cells, resting mast cells, eosinophils, and neutrophils, while naive B cells, CD8 T cells, activated memory CD4 T cells, and M1 macrophages decreased (Figure 7C). Additionally, there was a significant positive correlation between CDI and monocytes (Figure 7D). Survival analysis based on the infiltration levels of these cells significantly associated with CDI (Figure 7E‐M) revealed a strong correlation between CD8 T cells, CD4 T cells, M1 macrophages, and survival prognosis.

### 3.8 Predictive analysis of CDI on SKCM responsiveness to immune therapy, chemotherapy, and targeted therapy

**FIGURE 8:**
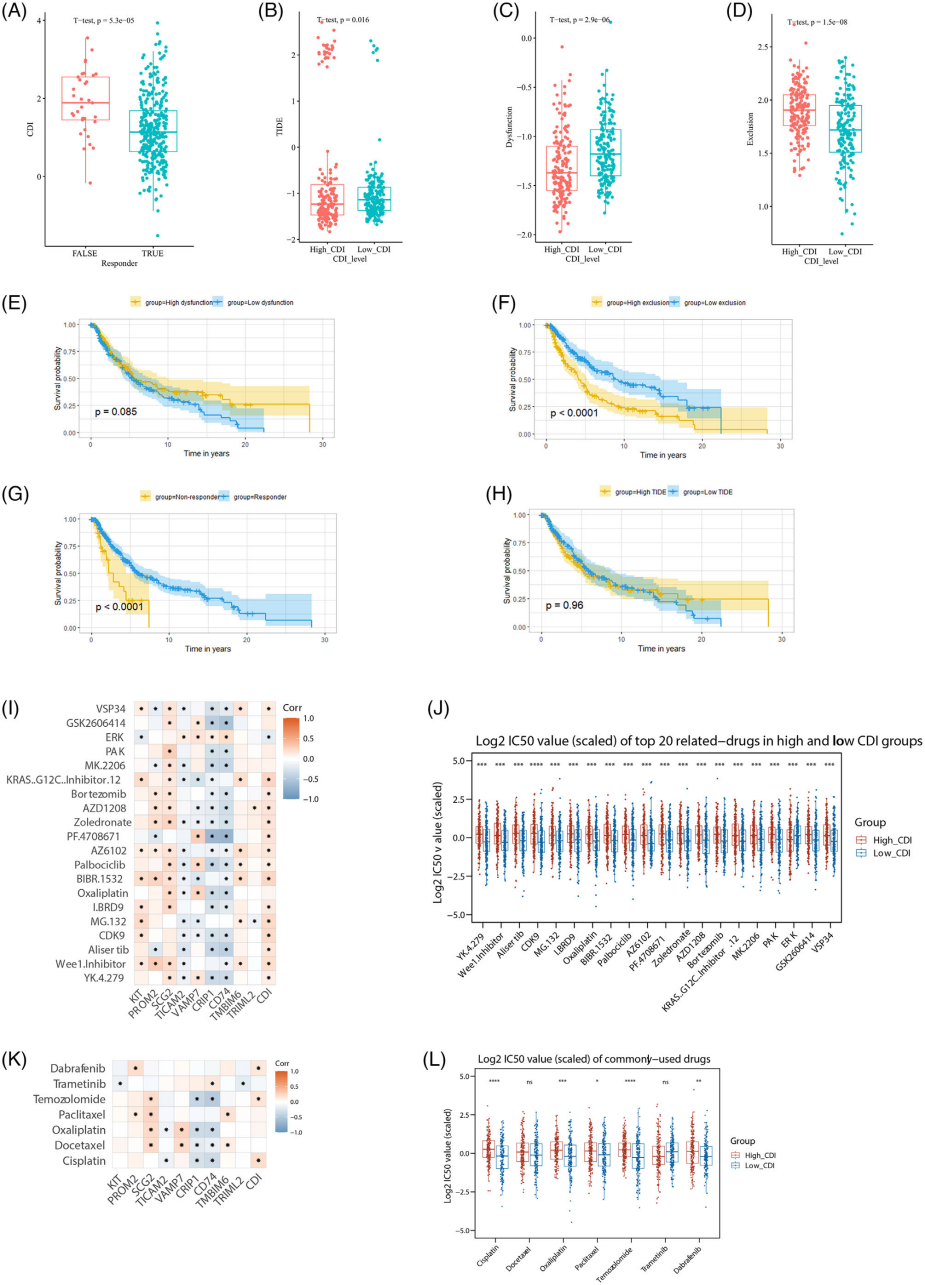
Predictive analysis of CDI on SKCM responsiveness to immune therapy, chemotherapy, and targeted therapy. (A) Differences in CDI levels between responders and non‐responders. (B–D) Differences in TIDE scores, dysfunction scores, and exclusion scores between patients in the high CDI group and low CDI group. (E–H) Kaplan‐Meier curves depicting survival among patients stratified by high/low dysfunction scores, high/low exclusion scores, responders versus non‐responders, and high/low TIDE scores. (I) Heatmap illustrating the correlation between the top 20 drugs with the greatest differences in drug sensitivity between the high CDI group and low CDI group. (J) Bar plot showing the IC50 values of the top 20 drugs with the greatest differences in drug sensitivity between the high CDI group and low CDI group. (K) Heatmap depicting the correlation between commonly used drugs for melanoma and CDI. (L) Bar plot showing the IC50 values of commonly used drugs for melanoma between the high CDI group and low CDI group. CDI, cell death index; SKCM, skin cutaneous melanoma; TIDE, tumor immune dysfunction and exclusion.

TIDE analysis revealed that patients predicted as responders to immune therapy exhibited relatively lower CDI levels, while non‐responders showed higher CDI levels (Figure 8A). Further investigation indicated significantly lower TIDE scores in the high CDI group (Figure 8B), significantly lower scores of Dysfunction (Figure 8C), and higher scores of Exclusion (Figure 8D). In survival analysis (Figure 8E‐8H), the responder status and Exclusion score were significantly associated with survival (*p* < 0.001).

In the sensitivity prediction of chemotherapy drugs and targeted therapy, 20 drugs displaying the most substantial variances in IC50 between the high CDI and low CDI groups were chosen. The association with CDI and differences between the high and low CDI groups are described in Figure 8I and Figure 8J. Additionally, among the seven commonly used drugs for SKCM out of the explored 198 drugs, their association with CDI and differences between the high and low CDI groups were analyzed (Figure 8K and Figure 8L). Several drugs such as dabrafenib, temozolomide, paclitaxel, oxaliplatin, and cisplatin showed a significant correlation with CDI levels.

## 4 DISCUSSION

In this study, we meticulously elucidated the expression patterns of PCD‐related genes in SKCM and their clinical implications. By developing a prognostic CDI based on PCD‐related gene expression, we effectively stratified SKCM patients into high and low‐risk categories, revealing significant differences in survival outcomes. Moreover, our findings elucidated the association between CDI and tumor stage, highlighting the clinical relevance of PCD dysregulation in SKCM progression. Functional enrichment analysis revealed distinct biological processes associated with high and low CDI groups, shedding light on the potential role of PCD dysregulation in immune modulation and tumor progression. Additionally, PPI analysis pinpointed crucial molecular networks associated with PCD dysregulation, thereby providing valuable insights into the underlying molecular mechanisms in SKCM pathogenesis. Overall, our study contributes to a deeper understanding of the PCD‐related molecular landscape in SKCM and its clinical implications, paving the way for the development of novel prognostic biomarkers and personalized therapeutic strategies tailored to individual patient needs.

In our research, we identified TMBIM6 as a pivotal gene associated with SKCM prognosis. This gene has garnered considerable attention owing to its multifaceted roles in tumorigenesis and cancer progression. TMBIM6 belongs to the transmembrane BAX inhibitor motif‐containing (TMBIM) family, which plays crucial roles in cellular homeostasis and apoptosis regulation. TMBIM6 is evolutionarily conserved and predominantly localized in the endoplasmic reticulum. It has been implicated in various cellular processes, including anti‐apoptotic, anti‐inflammatory, and anti‐oxidative functions.^37^ Moreover, TMBIM6 expression has been noted to vary across diverse tissues and developmental stages, suggesting its potential involvement in tissue homeostasis and developmental processes. Research has highlighted the significance of TMBIM6 in cancer biology. For instance, in breast cancer, TMBIM6 has been shown to modulate cell death pathways and promote cancer invasiveness.^38^ Similarly, in non‐small cell lung cancer, TMBIM6 expression was found to correlate with tumor growth and metastasis, indicating its potential as a prognostic biomarker and therapeutic target.^39^ In addition, studies in neonatal hypoxic‐ischemic rat models have demonstrated the neuroprotective role of TMBIM6, suggesting its involvement in regulating apoptosis and cell death pathways.^40^ Furthermore, miR‐302d‐3p‐mediated regulation of TMBIM6 has been implicated in breast cancer cell viability, migration, and apoptosis, highlighting the potential therapeutic implications of targeting TMBIM6 and its associated downstream signaling pathways.^41^ These findings collectively underscore the significance of TMBIM6 in cancer progression and advocate for its potential utility as a diagnostic biomarker and therapeutic target across various cancer types. Further elucidation of the molecular mechanisms governing TMBIM6's functions holds promise for the development of novel therapeutic strategies for cancer treatment.

The immune microenvironment, comprising a complex network of immune cells and their interactions, plays a critical role in the development and progression of SKCM. Immune cells, including lymphocytes, macrophages, dendritic cells, and others, contribute to tumor surveillance, modulation of anti‐tumor immune responses, and response to immunotherapy. Immunotherapy, notably immune checkpoint inhibitors targeting PD‐1 and CTLA‐4, has revolutionized the treatment paradigm of SKCM by augmenting anti‐tumor immunity and improving patient outcomes. In this study, we observed significant alterations in the immune microenvironment of SKCM, characterized by varying infiltration of macrophages and T cells in patients with different levels of CDI. M1 macrophages, with their potent anti‐tumor properties and ability to induce cytotoxic responses, were associated with favorable prognosis in SKCM patients. These findings underscore the pivotal role of macrophages in orchestrating anti‐tumor immune responses within the SKCM microenvironment. Targeting immune pathways associated with the recruitment and functionality of macrophages may represent a compelling therapeutic approach for improving outcomes in SKCM patients. Tumor‐associated macrophages (TAMs) have emerged as a promising therapeutic target in the management of melanoma. TAMs are abundantly present within the tumor microenvironment,^42^, ^43^ and predominantly associated with adverse clinical outcomes in cancer patients.^44^ Consequently, the colony‐stimulating factor 1 receptor (CSF1R) signaling pathway has garnered increasing attention as a therapeutic target. It has been reported that CSF1/CSF1R plays a pivotal role in the proliferation, differentiation, and functionality of macrophages.^45^ Hence, the inhibition of the CSF1R signal is anticipated to hinder the TAMs' function. A number of inhibitors and neutralizing antibodies targeting CSF1R have been developed and tested to examine their relevance in clinical therapy based on CSF1R inhibition.^46^ Numerous preclinical and clinical studies have demonstrated that inhibiting CSF1R leads to a reduction in TAMs and microglia.^47^ Inflammation is the consequence of the innate immune response to disturbed tissue homeostasis. Chronic inflammation is a common feature of cancer and plays a crucial role in promoting tumor development and progression. Both macrophages and tumor cells are characterized by their ability to produce pro‐inflammatory cytokines and inflammatory mediators, thereby sustaining tumor cell proliferation and survival, immune evasion, angiogenesis, metastasis, and chemotherapy resistance.^48^, ^49^ Accordingly, the inhibition of inflammation promoting cancer is anticipated by targeting key mediators and/or regulatory factors (such as NF‐κB and STAT3) of inflammatory pathways and cytokines (e.g., IL‐1, TNF, and IL‐6). Unfortunately, few antibodies/inhibitors have demonstrated anti‐tumor activity in preclinical studies. Thus, only a limited number of candidate drugs are currently under investigation in early clinical trials.^50^ The primary challenge in targeting inflammation is how to develop selective anti‐inflammatory approaches without compromising anti‐tumor immunity. Comprehending the fundamental constituents of the tumor microenvironment, such as fibroblasts and macrophages, may aid in the discovery and advancement of novel therapeutics targeting the tumor microenvironment. Importantly, the high heterogeneity of the tumor microenvironment has enabled the research and pharmaceutical industries to develop reliable biomarkers to guide therapy targeting the tumor microenvironment. In conclusion, establishing combination therapies aimed at maximizing therapeutic efficacy is envisioned to benefit a broader spectrum of patients. Overall, our study highlights the importance of understanding and harnessing the immune microenvironment, particularly the role of macrophages and immune reactions, in the management of SKCM.

While this study offers valuable insights, it is essential to acknowledge its limitations. While the validation sets utilized in this study are of modest size, their limited scale may influence the robustness of our findings. Despite efforts to internally and externally validate the prognostic CDI, larger validation cohorts with diverse patient demographics are needed to enhance the model's reliability. Additionally, while leveraging datasets from TCGA and GEO, it is important to acknowledge their inherent limitations, including subset representation and potential biases in patient selection and data preprocessing. Lastly, it is noted that the in vivo investigation is lacked in this study, and the IHC analysis is limited by the number of samples, as the available data in the Human Protein Atlas database may not comprehensively represent the entire spectrum of SKCM patients. Further investigation and validation through experimental studies are necessary to fully understand these complexities.

## 5 CONCLUSION

In conclusion, our study elucidates the prognostic significance of PCD‐related genes in SKCM. We have established and validated a prognostic CDI based on the expression of PCD‐related genes, and demonstrated its efficacy in stratifying SKCM patients into high and low‐risk groups with significant disparities in survival outcomes. Moreover, CDI shows promise as a predictive biomarker for survival and responsiveness to therapy, underscoring its potential clinical utility in guiding personalized treatment strategies for SKCM patients. These findings contribute to advancing our comprehension of SKCM pathogenesis and highlight the significance of PCD dysregulation in tumor biology.

## CONFLICT OF INTEREST STATEMENT

The authors have no conflicts of interest to declare.

## Data Availability

All data generated or analyzed during this study are included in this published article.
